# Development of rat female genital cortex and control of female puberty by sexual touch

**DOI:** 10.1371/journal.pbio.2001283

**Published:** 2017-09-21

**Authors:** Constanze Lenschow, Johanna Sigl-Glöckner, Michael Brecht

**Affiliations:** 1 Bernstein Center for Computational Neuroscience Berlin, Humboldt-Universität zu Berlin, Berlin, Germany; 2 NeuroCure Cluster of Excellence, Humboldt-Universität zu Berlin, Berlin, Germany; University of California San Diego, United States of America

## Abstract

Rat somatosensory cortex contains a large sexually monomorphic genital representation. Genital cortex undergoes an unusual 2-fold expansion during puberty. Here, we investigate genital cortex development and female rat sexual maturation. Ovariectomies and estradiol injections suggested sex hormones cause the pubertal genital cortex expansion but not its maintenance at adult size. Genital cortex expanded by thalamic afferents invading surrounding dysgranular cortex. Genital touch was a dominant factor driving female sexual maturation. Raising female rats in contact with adult males promoted genital cortex expansion, whereas contact to adult females or nontactile (audio-visual-olfactory) male cues did not. Genital touch imposed by human experimenters powerfully advanced female genital cortex development and sexual maturation. Long-term blocking of genital cortex by tetrodotoxin in pubescent females housed with males prevented genital cortex expansion and decelerated vaginal opening. Sex hormones, sexual experience, and neural activity shape genital cortex, which contributes to the puberty promoting effects of sexual touch.

## Author summary

We recently identified the somatosensory representation of rat genitals; remarkably, this cortical region—genital cortex—is sexually monomorphic, despite the marked sexual dimorphism of external genitals in rats. Most intriguing was the observation that genital cortex doubles in size during puberty. In order to shed light on this unusual expansion, we studied genital cortex development and sexual maturation in the female rat. We first showed that sex hormones are likely to cause the pubertal expansion of genital cortex. Next, we examined whether sexual experience affects the development of female genital cortex. Raising females together with adult males advanced genital cortex expansion, but cohousing with adult females or exposure to nontactile male cues was not sufficient to drive genital cortex growth. Surprisingly, artificial genital touch led to an early onset of female puberty and growth of genital cortex. In line with this finding, we find that if genital cortex activity is blocked, the advancing effects of adult males on puberty and genital cortex growth are inhibited. Together, our results point to an important role of genital cortex in the puberty-promoting effects of sexual touch.

## Introduction

Early analysis of the development of visual cortex by Hubel and Wiesel focused on binocular interactions and showed that both anatomy and physiology of ocular dominance is plastic and shaped in a visually-driven, activity-dependent process [1]. In a similar vein, the development of circuits in the somatosensory cortex (S1) was studied. It was recognized early on that precise topographic (barrel) representation of the whisker pattern in cortical input layer 4 [2] is imposed by peripheral inputs and has an early and brief critical period, after which it can no longer be changed [3]. Subsequent work identified neurogenetic mechanisms of cortical pattern formation [4]. With few exceptions, [5] most of the work provided little evidence for neural activity dependent processes in the development of S1. From work on the barrel cortex a consensus emerged that cortical input layer 4 has an earlier critical period and shows less plasticity than other cortical layers [6]. In the light of aforementioned results, the recently described developmental pattern of cortical input layer 4 of genital S1 was very surprising. Specifically, layer 4 of rat genital cortex showed a major expansion during puberty [7]. We wondered if this very late development of genital cortex has implications for the role of activity in cortical development and might even allow early sexual experience to impact on cortical development.

We were also interested in the relationship between somatosensory genital cortex development and female sexual maturation. In rodents, puberty has been intensely studied and much is known about changes in subcortical structures, such as the hypothalamus, that are associated with puberty [8]; there is, however, virtually no information available about changes in cortical circuits during puberty. A host of studies in a wide variety of species came to the conclusion that sexual maturation is not a purely age-dependent process but is also under social control. In mice, studies on female sexual development showed puberty advancing effects of male (primer) pheromones [9] and puberty delaying effects of adult female pheromones [10]. Most of the research on the social control of puberty has focused on pheromones [10] and identified the vomeronasal organ as key mediator of such effects in mice [11]. There is also evidence, however, that tactile interactions and sexual touch can powerfully influence sexual development. For example, the extreme reproductive suppression in the eusocial mole rat appears to be mediated by tactile rather than olfactory cues [12]. In a landmark study on mice, Bronson and Maruniak [13] showed that the puberty advancing effects of male pheromones alone are small. Bronson and Maruniak [13] suggested that the advance in female puberty caused by adult males results from a synergistic interaction of pheromones and tactile stimulation; Bronson and Maruniak [13] also ruled out a role of visual and auditory cues. The underlying neural mechanisms and pathways mediating male touch induced advance of female puberty are still unknown.

In primates, the evidence for pheromonal effects on puberty is rather mixed [14]. Sexual touch, on the other hand, is strictly regulated in most human cultures and this is particularly true during development. It has also become painfully clear that sexual abuse and inappropriate sexual contact during development have long-lasting detrimental consequences [15,16]. Presumably the long-lasting problems from inappropriate sexual contact during development reflect brain changes resulting from sexual experience. Remarkably, structural brain imaging in humans with a history of sexual abuse identified a thinning of putative human genital cortex, as a cortical consequence of childhood sexual abuse [17].

Here we ask the following questions: (1) Are sex hormones required for the pubertal expansion of female genital cortex? (2) What are the structural events underlying genital map changes? (3) Does sexual experience affect the development of female genital cortex? (4) Does activity in female genital cortex affect female puberty and does it mediate the female puberty advancing effects of male sexual touch?

## Results

### Pubertal expansion, but not maintenance of an adult size genital cortex, requires sex hormones

**Fig 1:**
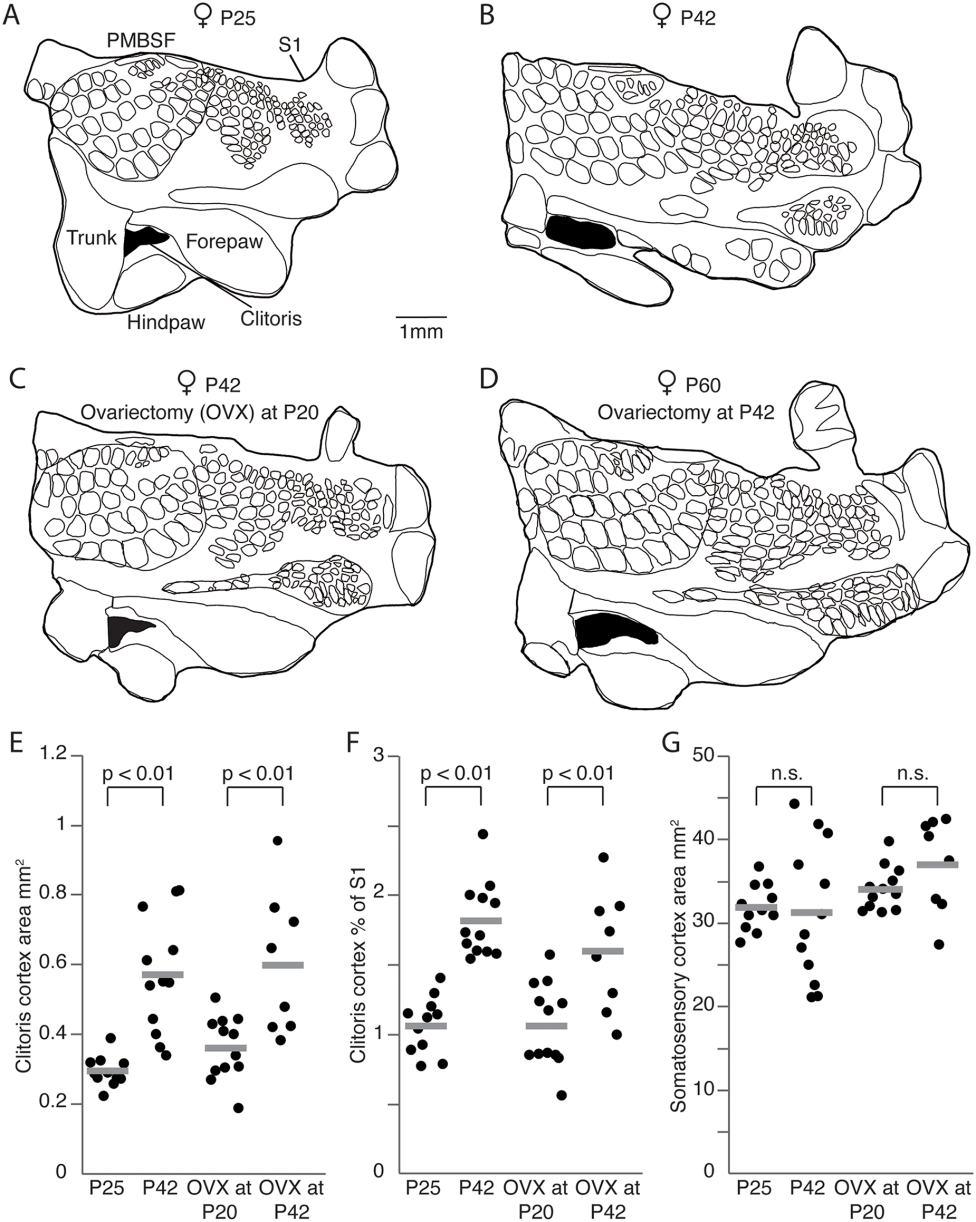
Pubertal expansion of genital cortex, but not its maintenance in adults requires sex hormones. (A) Outline of a somatosensory cortex (S1) map obtained from a female, aged postnatal day (P)25. Genital cortex is labeled in black. (B) Same as (A), but for an adult female (P42). Note the remarkable size difference of the genital cortex compared to the P25 female. (C) Outline of a S1 map from the brain of an adult female (P42), in which the ovaries were removed at P20. (D) Same as (B), but for an adult female (P60), in which the ovaries were removed at P42. The area of the genital cortex is similar to a nontreated adult female (B) and is bigger than in female rats ovariectomized before puberty (C). (E) Absolute area of clitoris in hemispheres of P25, P42 females, and females which were ovariectomized at either P20 or P42. (F) Fraction of genital cortex of the entire S1 in hemispheres of P25, P42 females, and females which were ovariectomized at either P20 or P42. Note that there is a substantial growth of the genital cortex between P25 and P42 animals. Female rats ovariectomized during prepuberty had smaller genital cortices than animals ovariectomized after puberty. (G) Absolute area of S1 in hemispheres of P25, P42, in prepuberty (P20) ovariectomized, and postpuberty (P42) ovariectomized female rats. See also S1 and S2 Figs, S1 Table, and S1 Data.

As a first step in our analysis we performed additional experiments to confirm our recently reported data on the growth of genital cortex during puberty [7]. To this end we analyzed the layer 4 cortical input maps in the S1 of 6 young (approximate postnatal day [P] 21) and 7 old (approximately P42) female rats. Specifically, we performed cytochrome oxidase staining, which reveals granular layer 4 regions (S1A–S1D Fig). We confirmed our earlier conclusions: whereas the clitoris representation in young prepubescent animals was rather small (Fig 1A), it appears to be twice as big in adult animals (P42, Fig 1B and 1E; *P* < 0.01, Student *t* test). In contrast, the size of the entire S1 did not differ significantly between young and old females (Fig 1G; *P* = 0.3, Student *t* test).

Next, we examined whether sex hormones play a role during the pubertal expansion of genital cortex. Hence, we ovariectomized females before (at P21) and after puberty (at P42). Twenty days after the ovariectomy animals were killed, brains were removed and tangential sections through the S1 were prepared and stained for cytochrome oxidase activity (S1C and S1D Fig). We obtained detailed anatomical maps of layer 4 from 7 prepubertal (Fig 1C) and 6 postpubertal (Fig 1D) ovariectomized females. Fig 1C shows a drawing of a complete S1 (thick outline) body map of a female aged P42, in which we removed the ovaries before puberty (at P21). The size of the cortical clitoris representation is small and comparable to the one of the young female, as depicted in Fig 1A. The somatosensory cortical map obtained from a female rat ovariectomized after puberty at P42 (Fig 1D) allows 2 observations: (1) the clitoris representation is much larger than the one of the female rat ovariectomized before puberty; (2) the size of the genital cortex is comparable to the genital cortex size of an adult female (Fig 1B). The population data (Fig 1E) suggest the same conclusions. The clitoris representation in females ovariectomized before puberty (mean genital cortex of S1 1.07% ± 0.09) was comparable to young females (1.07% ± 0.06) but significantly (*P* < 0.01, Student *t* test) smaller than the genital cortex size in animals, which were ovariectomized after puberty (1.6% ± 0.15; Fig 1F). Comparison of the genital cortex to the posteromedial barrel subfield (PMBSF), instead of the whole S1, led to the same results (S2A–S2C Fig) with no difference in the variability (S1 Table).

We conclude that sex hormones are required for the pubertal expansion of female genital cortex but not for the maintenance of an adult size female genital cortex.

### Systemic estrogen treatment accelerates genital cortex growth and advances female puberty

**Fig 2:**
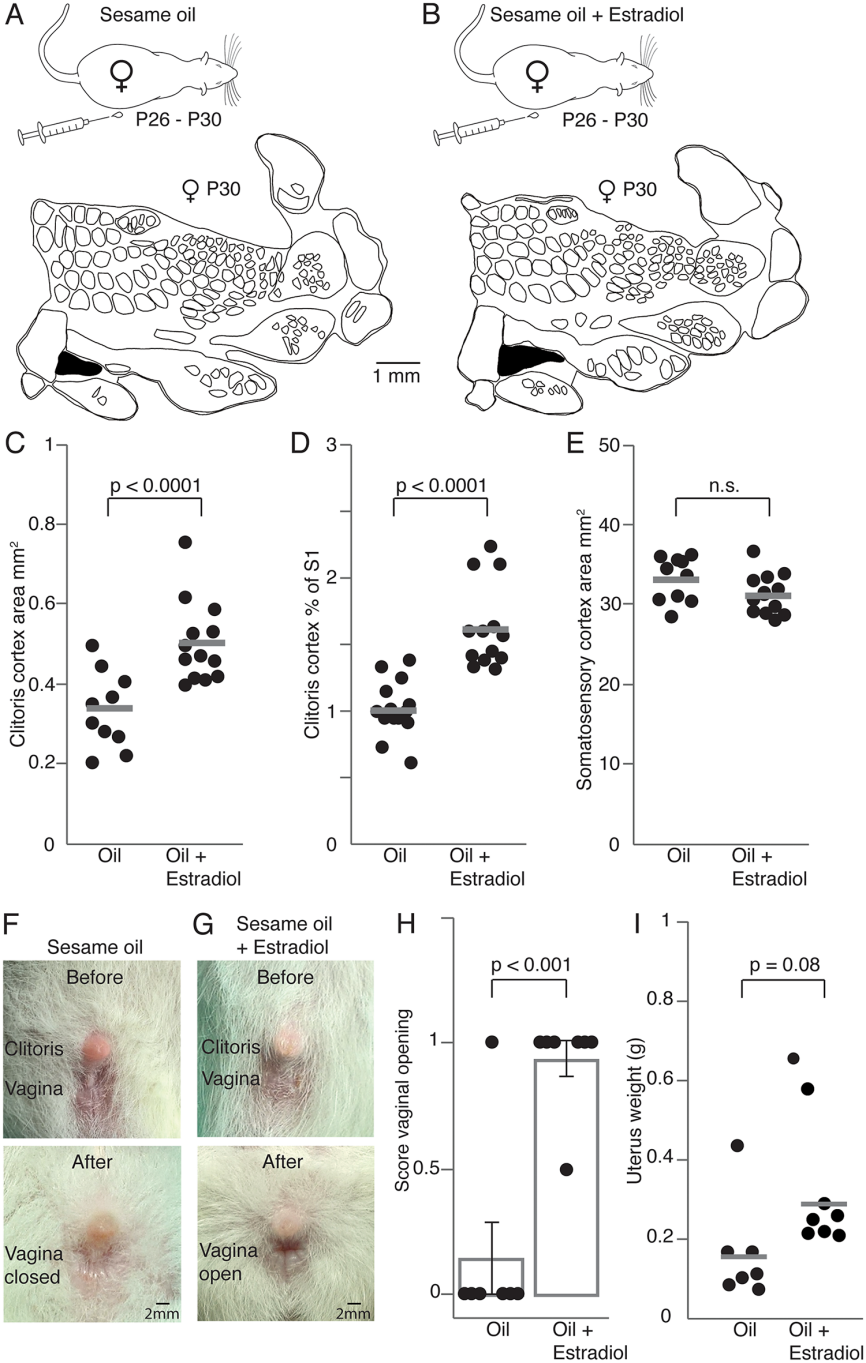
Systemic estradiol application drives genital cortex growth and advances puberty. (A) Upper panel: Control rats received daily subcutaneous injection of sesame oil for 5 days (postnatal day [P]26–P30). Lower panel: Outline of a somatosensory cortex (S1) map from the brain of a P30 female treated only with oil. The genital cortex is labeled in black. (B) Same as (A), but prepubescent rats were injected with sesame oil containing estradiol. (C) Absolute area of the clitoris representation in hemispheres of P30 females, which received daily injections (over 5 days) of either sesame oil alone or sesame oil containing estradiol. The genital cortex appears bigger in the estradiol group. (D) Same as (C), but the fraction of genital cortex of the entire S1 is plotted. (E) Absolute area of S1 in hemispheres of animals that received either daily oil or estradiol injections. There is no difference between the 2 groups. (F) Pictures of clitoris and vagina, before and after sesame oil treatment. Note that the vagina stays closed at P30 when animals were injected with sesame oil alone. (G) Same as (F), but for the vagina of estradiol treated animals. The vagina is already open at P30 (lower picture). (H) Mean scores for vaginal opening. A score of 0 represents a closed vagina, whereas a score of 1 stands for an opened vagina. The intermediate state (between open and closed vagina) has a score of 0.5. The vagina stayed closed in almost all control animals (treated with oil) whereas the majority of estradiol animals, showed an open vagina at P30. Each dot represents the score of vaginal opening for one animal. (I) Uterus weights of oil and estradiol treated animals. The uterus in estradiol treated animals is heavier, although this difference was not significant. Each dot represents the uterus weight from one animal. See also S3 Fig and S1 Data.

A strong impact of sex hormones on the onset of puberty has been reported using systemic estradiol injections during prepuberty [18]. We wondered to what extent systemic estrogen treatment can accelerate genital cortex growth. Female rats enter puberty typically at the age of P34 to P38 [19]. On that account and based on the study by Ramirez and Sawyer [18], we chose to treat females aged P26 for 5 days with estrogen and to test a possible effect on genital cortex at P30. Prepubescent females (P26) were injected over 5 days with either in sesame oil dissolved estradiol (Fig 2A, upper panel; *n* = 7 animals; 0.05μg estradiol, dissolved in 0.4 ml of sesame oil, per 100 g body weight) or with sesame oil alone (Fig 2B, upper panel; *n* = 7 animals; 0.4 ml of sesame oil, per 100 g body weight). During the fifth day of treatment animals were killed and cortical hemispheres were flattened, tangentially sectioned and processed for cytochrome oxidase activity (S3A and S3B Fig). In order to obtain cortical body maps, granular somatosensory regions were reconstructed through serial sections. To exclude experimenter biases cortical maps were drawn and analyzed by an experimenter blind to the condition estradiol versus oil treated animals.

The lower panel in Fig 2A shows a cortical map obtained from an oil-treated female. A cortical map obtained from a female, which was injected with estradiol, is shown in the lower panel of Fig 2B. The size of the clitoris representation is much larger in the estrogen treated female. Population data for all hemispheres confirm this result. The absolute area of genital cortex (Fig 2C; mean absolute area in oil injected animals was 0.33mm^2^ ± 0.025 and in estradiol injected animals 0.5mm^2^ ± 0.028; *P* < 0.001, Student *t* test) and the mean percentage of genital cortex of S1 (Fig 2D; mean genital cortex of S1 in estradiol treated animals was 1.6% ± 0.09 and in sesame oil injected animals 1.01% ± 0.06; *P* < 0.0001, Student *t* test) was significantly greater in estradiol injected animals compared with control animals (oil treated). The size of S1 was not different (Fig 2E; mean area of S1 in oil treated animals was 32.9mm^2^ ± 0.76 and in estradiol treated animals 31.2mm^2^ ± 0.68; *P* = 0.1, Student *t* test).

We also assessed parameters indicative of sexual maturation and the onset of puberty. Thus, we evaluated vaginal opening (an open vagina is typically seen in females, which have entered puberty, [18]) and the weight of the uterus, which is heavier in females after puberty [18]. Accordingly, we documented vaginal opening before, during and after the daily injections. In Fig 2F, 2 photographs show the clitoris and vagina of an oil treated female before the beginning of daily injections (P25, upper panel) and afterwards (P30, lower panel). Vagina and clitoris for an estrogen treated animal are shown in Fig 2G, respectively. Whereas the vagina of the oil treated female was still closed at P30, the estradiol injected animal shows an open vagina at this stage (Fig 2G, lower panel), indicating that the onset of puberty took place. To quantify vaginal opening, we gave the following scores for every vaginal opening stage (Fig 2H): a score of 0 points to a closed vagina, whereas a score of 1 indicates an open vagina. A score of 0.5 represents the intermediate state, during which the vagina was about to open. The majority of oil treated females (6 out of 7) showed a closed vagina at P30, whereas most of the estrogen injected animals displayed an open vagina (6 out of 7; *P* < 0.001, Mann–Whitney U test). We also removed and weighed the uteri. On average, the oil-treated animals had lighter uteri (0.15g ± 0.07, Fig 2I) compared to animals, which received daily estradiol injections (0.29g ± 0.08), but this difference did not reach significance (*P* = 0.08, Student *t* test). Taken together these data suggest that systemic estradiol accelerates genital cortex growth and advances the onset of puberty.

### Genital cortex growth is due to the invasion of dysgranular cortex by putative genital thalamic afferents

Which cellular changes underlie the unusual genital cortex expansion during puberty? To answer this question, we first stained alternating tangential sections of flattened cortices for cytochrome oxidase activity (S4A Fig) and with vesicular glutamate transporter 2 (VGluT2) antibodies (S4B Fig). While cytochrome oxidase reports constitutive layer 4 metabolic activity, VGluT2 is expressed in thalamocortical terminals [20] and, hence, labels thalamic afferents. S4A Fig (upper panel) shows a tangential section through S1 of a young animal (P14) stained for cytochrome oxidase activity and in S4B Fig (upper panel) the subsequent section is shown labeled for VGluT2. The labeled structures have the same layout and size, as confirmed by quantitatively analyzed drawings (S4A and S4B Fig, lower panels). In old animals, alternating staining for cytochrome oxidase activity and with VGluT2 also led to completely overlapping results. We conclude from these observations that pubertal genital cortex growth leads to an expansion of the cortical area innervated by thalamic afferents.

**Fig 3:**
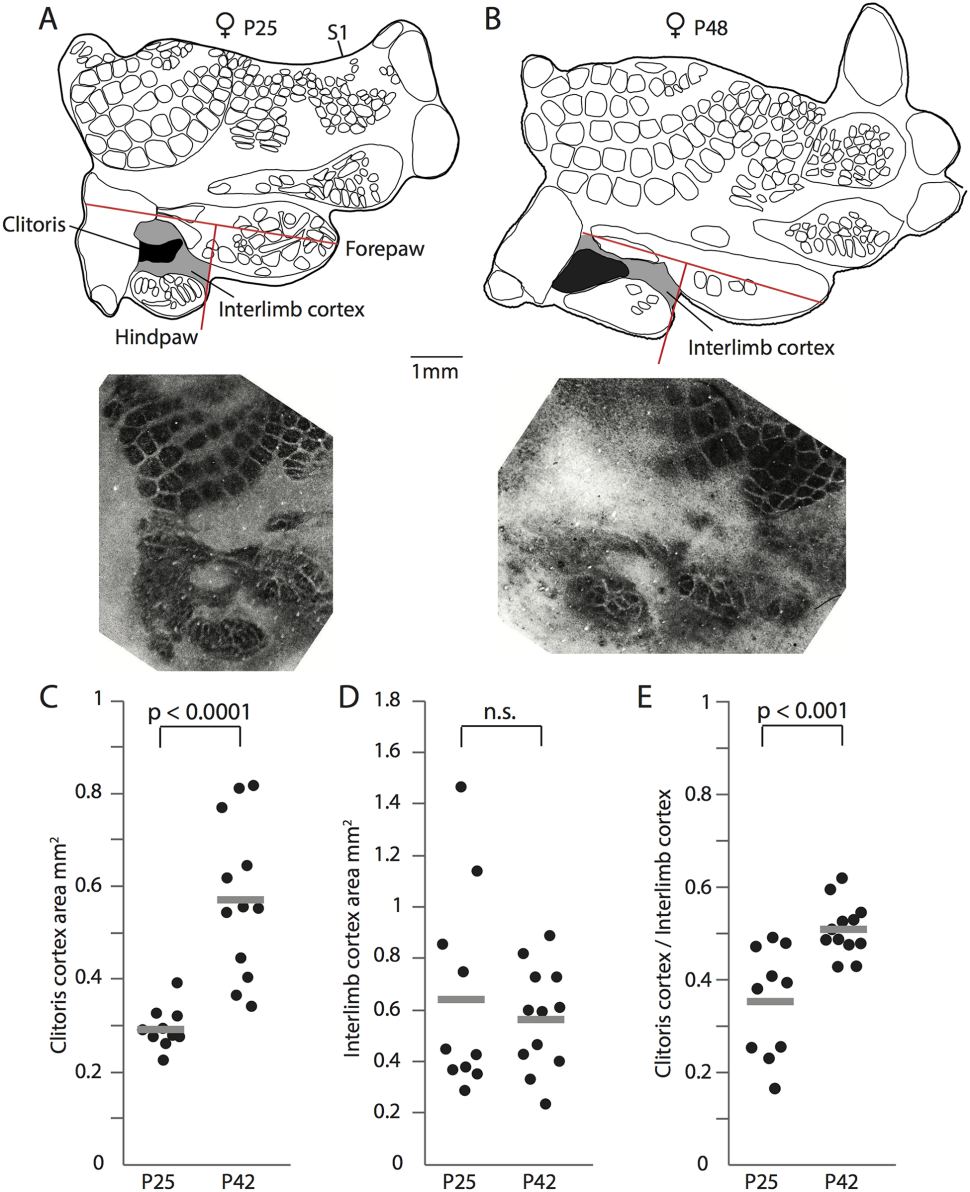
Genital cortex growth is due to the invasion of dysgranular territories. (A) Upper panel: Map of somatosensory cortex (S1) obtained from a female aged postnatal day (P) 25. Genital cortex is labeled in black. The interlimb cortex is marked in grey. The upper red lines marks the forepaw axis. The lower red line is rectangular to the forepaw axis, terminates at the anterior end of the hindpaw representation, and was used to demarcate interlimb cortex. Lower panel: Corresponding for cytchrome c stained tangential section, which shows best the clitoris area. (B) Same as (A), but for an animal aged P48. Note that the interlimb cortex area is smaller than in the young animal and that the clitoris representation is larger. (C) Absolute area of the clitoris representation in hemispheres of young (P25) and old (P42) animals. The clitoris representation almost doubles in size. (D) Same as (C), but the absolute area of the interlimb cortex is plotted. The interlimb cortex is slightly smaller in area in old animals than in young animals. (E) The ratio of the genital cortex to the interlimb cortex increases after puberty in P42 animals. See also S4 Fig and S1 Data.

Next, we asked, how the genital cortex is able to increase its thalamically innervated area so drastically. Does the genital cortex expand like a balloon, pushing the neighboring cortex aside? Alternatively, do genital afferents invade neighboring territories? To address this issue, we analyzed S1 maps from young and old animals in more detail (Fig 1A and 1B). Specifically, we measured the space between fore- and hindpaw, as shown in Fig 3A and 3B, and named it interlimb cortex. The interlimb cortex (grey zone) is dysgranular in structure (i.e., does not have a distinct layer 4) and is larger in the map of a young animal (P25; Fig 3A, upper panel) than in the one of an old animal (P48; Fig 3B, upper panel). This size decrease is very surprising, as S1 grows overall by approximately 15% during puberty (note that this growth in S1 did not reach significance between young and old females; Fig 1). Cytochrome oxidase stained sections (Fig 3A and 3B, lower panels) confirm this result. Whereas the absolute genital cortex area almost doubles in size from P21 to P42 (Fig 3C), the absolute interlimb cortex area slightly decreases (Fig 3D; mean interlimb cortex area in S1 maps of young animals was 0.64mm^2^ ± 0.06 and in old animals 0.56mm^2^ ± 0.1). Interestingly, the ratio of the genital cortex to the interlimb cortex significantly increases during puberty (Fig 3E; mean ratio of genital cortex to the interlimb area in young animals was 0.35 ± 0.035 and in old animals 0.51 ± 0.017; *P* < 0.001, Student *t* test). The simultaneous increase of genital cortex and decrease of interlimb cortex suggest that the pubertal genital cortex growth reflects an invasion of dysgranular interlimb cortex by putative genital thalamic afferents.

### Genital cortex growth in prepubescent females is accelerated by tactile cues from males

**Fig 4:**
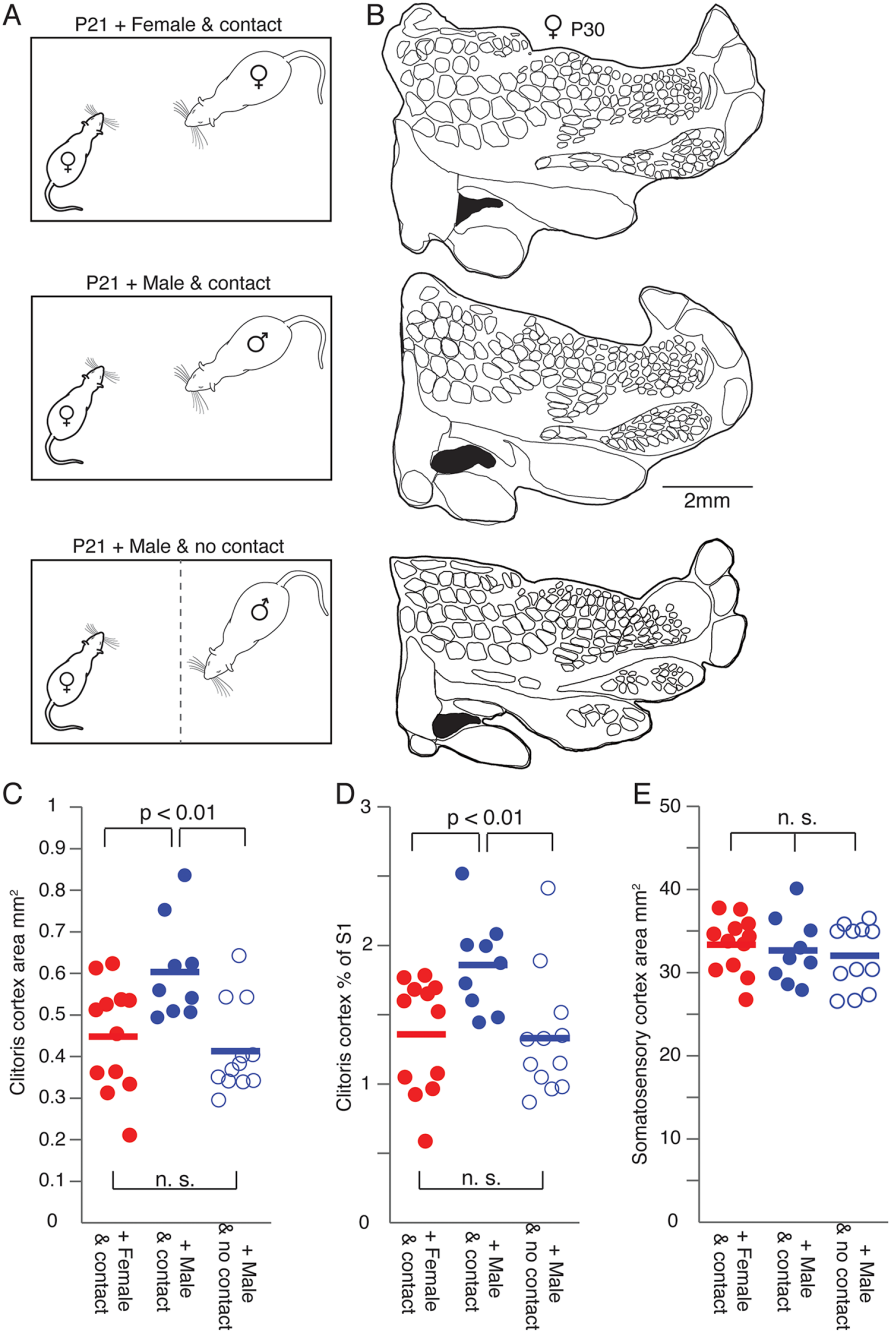
Genital cortex growth in prepubescent females is accelerated by tactile cues from males. (A) Prepubescent animals (postnatal day [P]21) were cohoused for 9 days with either an adult, sexually experienced female (upper panel) or a sexually experienced adult male (middle panel). Whereas tactile contact was allowed in both of these groups, the third group of prepubescent rats was only exposed to olfactory (by a daily exchange of bedding between the cage compartments), visual, and auditory cues of a sexually experienced adult male (lower panel). (B) Upper panel: Outline of a somatosensory cortex (S1) map from the brain of a P30 female cohoused with a sexually experienced adult female. Genital cortex is labeled in black. Middle panel: Map from a brain of a P30 female cohoused with a sexually experienced adult male. Lower panel: Outline of a S1 map obtained from a P30 female exposed to olfactory, visual, and auditory cues but not tactile cues from a sexually experienced adult male. (C) Absolute area of genital cortex in hemispheres of P30 females cohoused with either a female (female and contact), a male (male and contact), or with a male without having tactile contact (male and no contact). Note that the size of genital cortex in the brains of animals who were cohoused in tactile contact with a male is significantly larger compared to the other 2 groups (female and contact, and male and no contact). (D) Same as (C), but the fraction of genital cortex of the entire S1 is shown. (E) Same as (C), but the absolute area of S1 is plotted. There is no difference between the 3 groups. See also S1 Data.

Although sex hormones have been identified as important players during the onset of puberty, there is also evidence that somatosensory stimuli (olfactory or tactile) can influence sexual development. Thus, we assessed if sexual experience affects the development of female genital cortex. To this end, we chose the following experimental paradigm, which is inspired by the seminal work of Bronson and Maruniak [13]. Prepubescent females (P21) were cohoused for 9 days with (1) sexually experienced females (Fig 4A, upper panel), or with (2) sexually experienced males (Fig 4A, middle panel), or with (3) sexually experienced males separated by a wire mesh (Fig 4A, lower panel). In the first 2 groups, the animals had direct contact to interaction partners. In the third group, animals were cohoused but had no tactile access to them because of a wire mesh dividing the cage (Fig 4A, lower panel). Importantly, bedding was swapped between the 2 cage compartments daily, such that females received full exposure to pheromonal cues. Females were also able to see and hear the adult male. At the age of P30 animals were killed. Brains were removed and tangential sections through layer 4 of S1 were prepared and stained for cytochrome oxidase activity. The experimenter reconstructing and analyzing cortical maps was again blind to the experimental condition.

In Fig 4B somatosensory cortical maps are shown for all 3 experimental conditions (upper panel: map obtained from a female cohoused with a female; middle panel: map from a female cohoused with a male; lower panel: map from a female, which sat together with a male, but with a wire-mesh separating the animals). Note that the cortical clitoris representation is the largest in the map of the female cohoused together with a sexually experienced male. In fact, the cortical clitoris representation (Fig 4C) and the fraction of clitoris of the entire S1 (Fig 4D) increased significantly more, when prepubescent females received male tactile cues (filled blue circles, mean genital cortex of S1 1.9% ± 0.1) compared to female tactile cues (filled red circles, mean genital cortex of S1 1.36% ± 0.1). Interestingly, pheromones and audio-visual contact alone were insufficient to drive genital cortex growth (open blue circle, mean genital cortex of S1 1.3% ± 0.1). A 1-way ANOVA also reported a significant difference between prepubescent females cohoused with either males, females, or males without having tactile contact (*P* = 0.01). The overall size of S1 (Fig 4E) did not differ between groups (*P* = 0.67, 1-way ANOVA). Note that the relative size of genital cortex of the young females, which were cohoused with sexually experienced males (mean genital cortex of S1 1.9% ± 0.1, Fig 4D filled blue circles), is similar to that of the adult females (mean genital cortex of S1 1.8% ± 0.1, Fig 1B), even though we killed these animals at P30, which would correspond to midpuberty group raised animals. These findings suggest, that male sexual touch strongly advances female genital cortex growth.

### Artificial genital touch drives genital cortex expansion and promotes female sexual maturation

**Fig 5:**
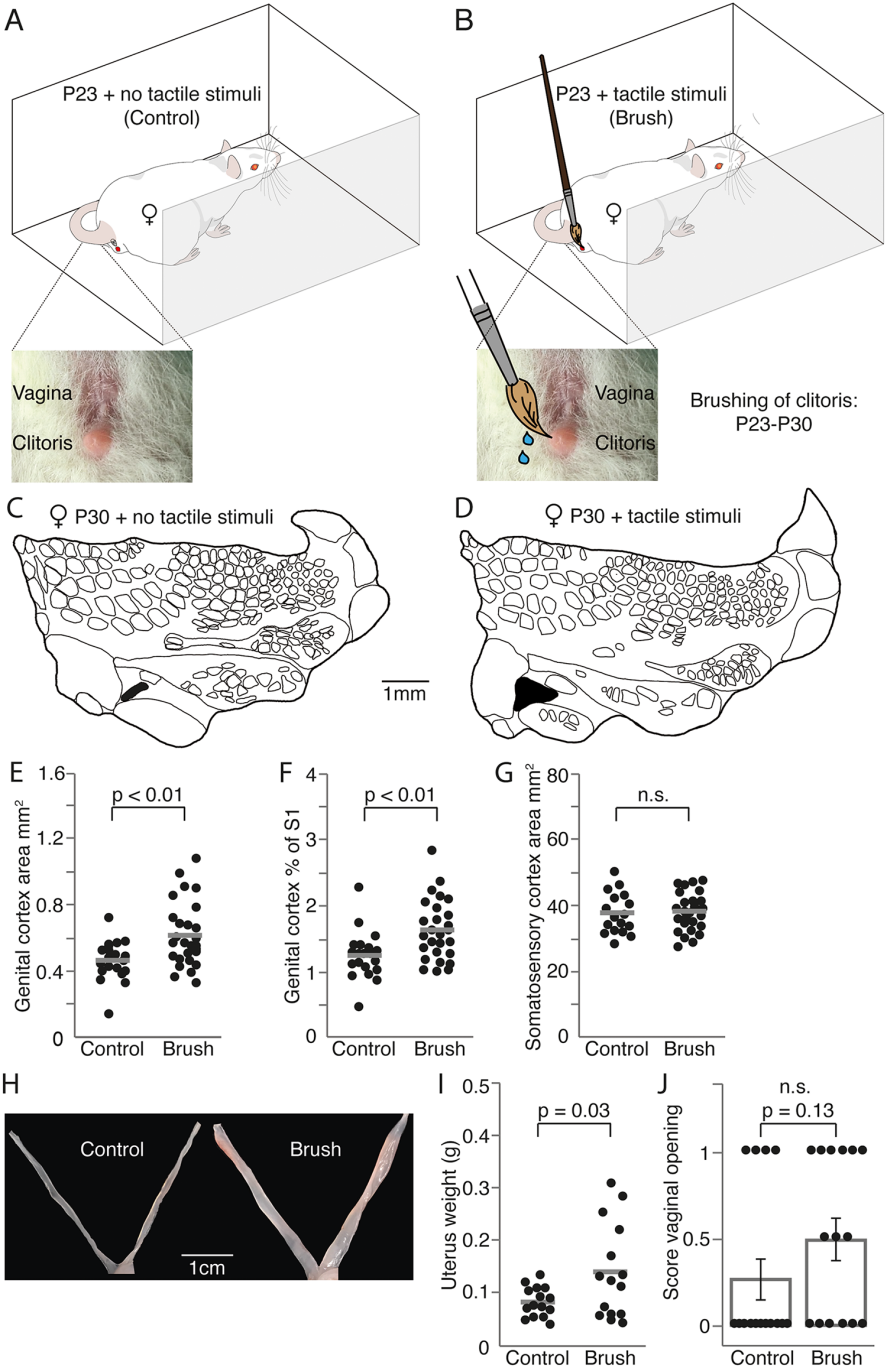
Artificial genital touch drives genital cortex expansion and promotes female sexual maturation. (A) Control, prepubescent female rats (postnatal day [P]23) were placed each day 3 times for 10 minutes in a box across 7 days without further treatment. (B) Artificial genital touch; prepubescent female rats (P23) were placed each day 3 times for 10 minutes in a box across for 7 days, and their clitoris and vulva were stroked with a lubricated brush by a human (female) experimenter. (C) Outline of a somatosensory cortex (S1) map from the brain of a P30 female who received the control treatment described in A. Genital cortex is depicted in black. (D) Same as (C), but example map stems from the brain of a P30 female, whose genitals were brushed as described in (B). (E), Absolute area of clitoris representation in hemispheres of P30 females, which underwent control (A) or artificial genital touch (B) treatment. (F) Same as (E), but the fraction of genital cortex of the S1 is plotted. (G) Same as (E), but the absolute area of S1 is shown for the 2 groups. There is no difference in S1 size. (H) Mean scores for vaginal opening for P30 females, which underwent control (A) or artificial genital touch (B) treatment. (I) Picture showing the uterus of a female, which underwent control (A) or artificial genital touch (B) treatment. (J) Uterine weights of females that underwent control (A) or artificial genital touch (B) treatment. See also S1 Data.

We were surprised to observe such big effects following cohousing with sexually experienced males, while male olfactory cues had no impact on genital cortex development. These observations made us wonder about the relative contributions of tactile and other cues on female sexual maturation and genital cortex development. What are the contact cues that drive genital cortex expansion and promote female sexual maturation? It has previously been shown, that gentle touch of female genitals creates conditioned place preference in female rats [21] and increases 50kHz range trill calls emitted by hormonally primed females [22]. Similarly, we set up an experiment, where prepubescent female rats (P23) were exposed to artificial genital touch (Fig 5). During a 10-minute session, animals were freely moving in a small U-shaped environment, while the female experimenter repeatedly touched the animals’ clitoris and vulva with a lubricated brush (artificial genital touch, Fig 5B). Control animals were placed in the same environment for 10 minutes without genital touch (Fig 5A). For 7 days, each animal completed 3 sessions distributed across the day. Animals were killed at P30 and S1 maps were reconstructed with the experimenter being blind to the condition. Control animals (Fig 5C) had a smaller genital cortex than animals, which received artificial genital touch (Fig 5D). Such differences in absolute (Fig 5E) and relative size of genital cortex (Fig 5F) were significant. The effect size was comparable to the differences observed between male and female touch in Fig 4; the size of the entire S1 was not different (Fig 5G). Artificial genital touch also affected female sexual maturation. While there was no significant change in vaginal opening (Fig 5J), the uteri were significantly heavier following artificial genital touch, as compared with control rats (Fig 5H and 5I).

### Blockade of neuronal activity prevents genital cortex growth and delays vaginal opening

**Fig 6:**
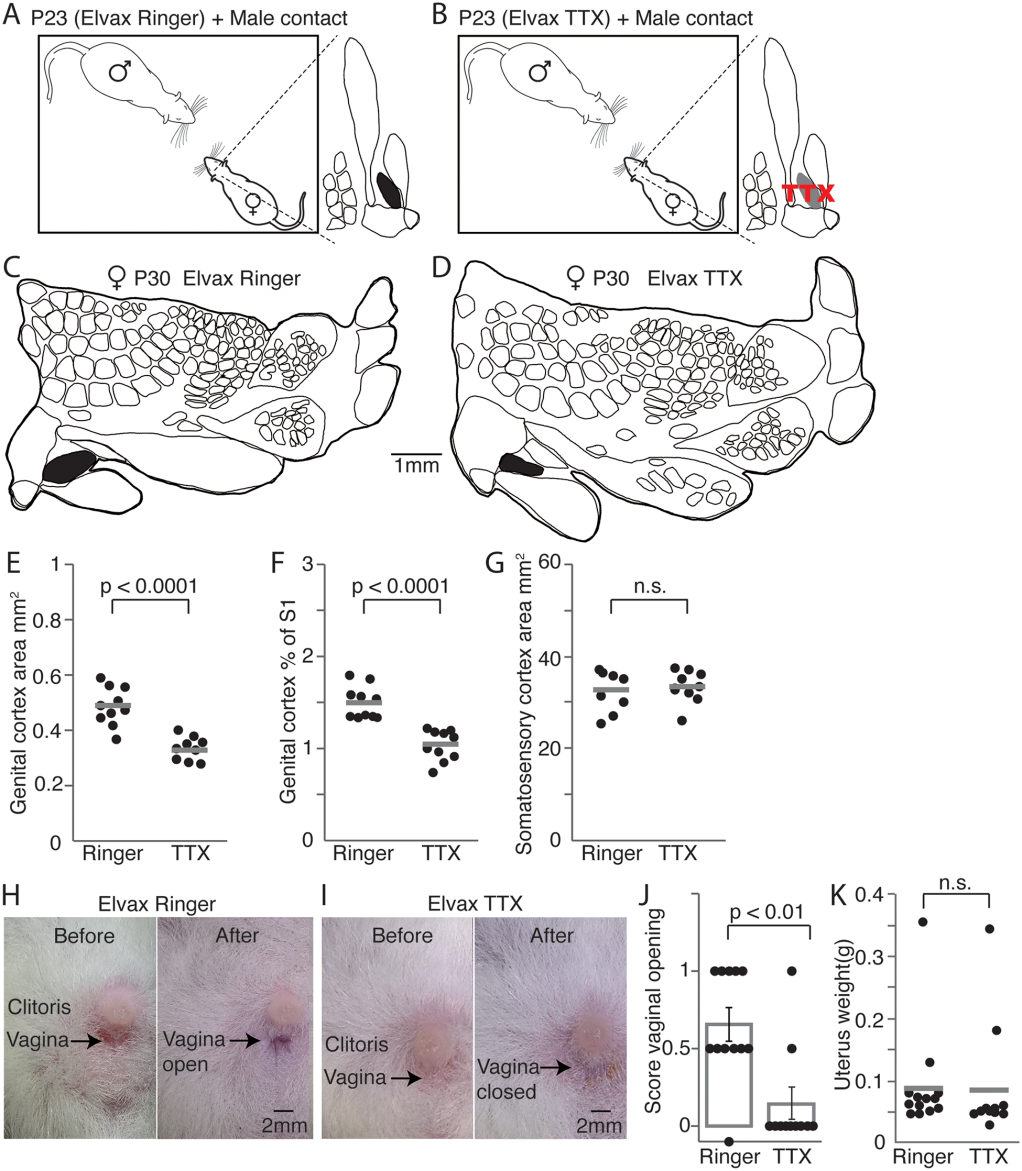
Blocking neuronal activity in genital cortex prevents area growth and delays female sexual maturation. (A) Prepubescent animals (postnatal day [P]23) received Ringer impregnated Elvax sheets above genital cortex and were cohoused together with a sexually experienced adult male for 7 days. (B) Same as (A), but Elvax implants released tetrodotoxin (TTX), which blocked neuronal activity in genital cortex. (C) Outline of a somatosensory cortex (S1) map from the brain of a P30 female, who received a ringer soaked implant above genital cortex and was cohoused with a sexually experienced adult male. Genital cortex is depicted in black. (D) Same as (C), but example map stems from the brain of a P30 female, whose genital cortex was blocked through a TTX containing implant. (E) Absolute area of clitoris representation in hemispheres of P30 females, which were cohoused with sexually experienced adult males and received beforehand either Elvax implants containing Ringer or TTX. Note that genital cortices in Ringer treated animals show significant larger growth compared with animals that received TTX implants above genital cortex. (F) Same as (E), but the fraction of genital cortex of the S1 is plotted. (G) Same as (E), but the absolute area of S1 is shown for the 2 groups. There is no difference in S1 size. (H) Pictures of clitoris and vagina taken before implanting animals with Ringer containing Elvax sheets (P23) and after cohousing them for 7 days with sexually experienced adult males. Note that the vagina was already open at P30. (I) Same as (H), but pictures show clitoris and vagina of an animal, who received a TTX-soaked Elvax implant above genital cortex. The vagina stayed closed when blocking genital cortex, while cohousing the prepubescent animals with a sexually experienced adult male. (J) Mean scores for vaginal opening. A score of 0 represents a closed vagina, whereas a score of 1 stands for an opened vagina. The intermediate state (between open and closed vagina) has a score of 0.5. The majority of females, which received a Ringer soaked implant above genital cortex, showed either an opened vagina or a half opened vagina, which was about to open after being cohoused for 7 days with sexually experienced adult males. In contrast, almost all animals implanted with TTX Elvax sheets had the vagina closed at P30. Each dot represents the score of vaginal opening for 1 animal. (K) Uterine weights of females with implanted Ringer or TTX Elvax sheets after being cohoused with sexually experienced adult males. No significant difference (*P* = 0.93, Student *t* test) was found between the groups. See also S1 Data.

In order to better understand the role of female genital cortex during puberty, we blocked genital cortex activity over a certain time during prepuberty (P23–P30). To do so, P21 animals received Elvax implants over the area of genital cortex. Elvax sheets were developed for the slow, gradual release of drugs [23]. Elvax sheets were either soaked with tetrodotoxin (TTX) (Fig 6B), which blocks voltage dependent sodium channels, or impregnated with Ringer, as a control condition (Fig 6A). After recovery, the implanted females were cohoused with sexually experienced males until the age of P30. S1 maps of the brains were obtained as described above. The experimenter reconstructing cortical maps was again blind to the experimental condition. A map from a control animal treated with ringer is shown in Fig 6C, while Fig 6D shows a cortical map obtained from a TTX treated female. In animals, in which genital cortex was blocked with TTX, the area of clitoris representation (Fig 6E; mean area in maps of control animals was 0.48mm^2^ ± 0.02 and in S1 of TTX treated animals 0.32mm^2^ ± 0.013; *P* < 0.0001, Student *t* test) and the relative size (Fig 6F) was smaller than in control animals. The area of S1 did not show any differences (Fig 6G; mean area of S1 in Ringer treated animals was 32.4mm^2^ ± 1.4 and in TTX implanted animals 33.1mm^2^ ± 1.1; *P* = 0.7, Student *t* test). The difference between hemispheres from control (mean genital cortex of S1 was 1.49% ± 0.05) and TTX treated animals (mean genital cortex of S1 was 1.02% ± 0.05) was marked (*P* < 0.0001, Student *t* test). These results immediately suggest 2 conclusions. First, the fact that there was a difference between control and TTX treated animals indicates that our TTX treatment was effective. Second, the data indicate that the pubertal expansion of genital cortex requires cortical activity.

Next, we wondered if an intact genital cortex is required for female sexual maturation. Therefore, we determined the status of puberty in control and TTX treated animals. Accordingly, pictures of vagina and clitoris were taken before implantation and after the behavioral experiment. Five out of 13 animals, which received a ringer soaked Elvax sheet above genital cortex, showed an open vagina at the end of the experiment. The vagina before (left panel) and after the experiment (right panel) for one of those females is shown in Fig 6H. In contrast, only 1 of the animals (*n* = 11), whose genital cortices were blocked with TTX, had an open vagina. Fig 6I shows the pictures of 1 TTX animal before (left panel) and after (right panel) the treatment. The scores of vaginal opening are plotted in Fig 6J. The vaginas were closed in 9 out of 11 animals treated with TTX, and we observed a vagina that was about to open in only 1 TTX treated animal. In contrast, only 2 out of 13 animals in the Ringer group had the vagina still closed at the end of the experiment. The uterine weights (Fig 6K) were not significantly different, however. Thus, genital cortex activity might relay effects of male sexual touch on vaginal opening in rat female puberty.

### The relation between relative genital cortex size, estrogens, and uterine weight

In order to obtain insight in the relationship between female sexual maturation and genital cortex development, we plotted genital cortex size against uterine weight in all experiments, in which we collected such data (S5 Fig). As shown in S5A Fig, there was no tight relationship between relative genital cortex size and uterine weight. Nonetheless, it was obvious that a large uterine weight (≥0.15 g) is rarely associated with a small (≤1%) relative genital cortex size. If one compared genital cortex size and uterine weight across specific experimental conditions (S5B Fig), it was noticeable that animals with TTX treatment of genital cortex (the experiment described in Fig 6) never had very large genital cortices, even for cases with a large uterus.

## Discussion

Our data confirm a major pubertal expansion of female genital cortex, which—unlike the maintenance of large genital cortex in adults—requires intact ovaries and sex hormones. The larger area of genital cortex in adults reflects an invasion of adjacent territories by thalamic afferents. Such changes in female genital cortex are advanced by sexual touch and require cortical activity. Most interestingly, blockade of genital cortex delays female sexual maturation, suggesting that genital cortex is the neural structure that mediates the puberty advancing effects of male sexual touch.

The expansion of layer 4 of genital cortex in puberty [7] is an unusual pattern in the development of layer 4 in S1. In contrast, the barrel representation is characterized by a brief directly postnatal critical period, after which the barrel pattern can no longer be changed [3]. Our data show that intact ovaries and, hence, presumably the sex hormones emitted by the ovaries are required for the pubertal expansion of genital cortex. The interpretation that sex hormones drive pubertal expansion is greatly strengthened by observations following the injection of estradiol, which mimics the pubertal expansion of genital cortex. The mechanisms, by which sex hormones, and in particular estrogens, drive pubertal expansion of genital cortex, are yet to be determined; both, a direct sex hormone effect on genital cortex and an indirect effect via growth stimulation of sex organs (i.e., the clitoris) by sex hormones are conceivable. Thus, it is possible that cortical changes follow similar mechanism, as described for the hippocampus, in which a rapid potentiation of excitatory synapses is achieved by estrogen [24] with different underlying mechanism in male and female rats [25]. From human studies, it is known that the hippocampus changes in size along the reproductive cycle with the biggest hippocampus being present when blood estrogen levels are highest [26].

The data discussed below indicate that at least some stage of cortical activity seems to be required for genital cortex expansion. The visualization of thalamic afferents by VGluT2 antibodies showed that cytochrome oxidase staining reveals maps completely congruent with thalamic innervation. Thus, the size increase of genital cortex in adults indicates that a larger cortical area is innervated by thalamic afferents. The detailed analysis of body maps in young and adult animals is strongly suggestive that the size increase of genital cortex results from an invasion scenario, in which putative genital afferents innervate neighboring dysgranular cortex.

Female genital cortex development is strongly affected by tactile sexual experience. Housing pubescent females with a sexually experienced adult male strongly advances the growth of genital cortex. Even though we killed such females at P30 (which corresponds in group-housed animals to midpuberty), they had genital cortices larger than average adult size (compare Figs 2D and 4D). This advancement of puberty was induced specifically by direct male contact and was not seen in animals cohoused but not touched by males, or in animals cohoused with females. In line with the idea that genital cortex is altered by sexual experience, we find that blockade of genital cortex prevents the pubertal layer 4 expansion of genital cortex. The requirement of experience and cortical activity for the appropriate development of adult somatotopy is similar to the requirement of cortical activity for the development of ocular dominance columns in the visual system [27]. It is notably different, however, from the development of the early postnatal development of the barrel pattern in S1, which occurs despite manipulation of peripheral [28] or cortical inputs [29]. Thus, genital cortex does seem to differ from barrel cortex not only in the timing of the critical period, but also the cellular mechanisms mediating genital cortex plasticity seem to differ from barrel cortex.

Whereas our blocking experiments show that genital cortex activity is crucial for the pubertal layer 4 expansion, it is questionable to what extent this plasticity is functionally meaningful. Decorticated animals are still able to mount and reproduce. However, subtle changes in the pattern of reproduction [30,31] are observed. Furthermore, somatosensory feedback from the penis was shown to be critical for the achievement of intromission, and somatosensory feedback from the preputial region is needed for the execution of copulatory thrusting [32]. Blocking genital cortex activity during the critical period of puberty may cause changes in the pattern of reproduction during adulthood, i.e., that the number of intromissions and time to ejaculation might be affected (in males); lordosis or place preference behavior could be affected in females.

As shown many decades ago, the social signals powerfully control the advance of puberty [33]. While it became clear that pheromonal stimuli mediated by the vomeronasal organ [11] contribute to the male induced advance in female puberty in mice, the hastening effects of puberty induced by male pheromones alone are minor. Bronson and Maruniak [13] suggested that tactile male cues synergistically with pheromones advance female puberty. Further work showed that androgenized female mice [34], but not castrated male mice [13], provide the tactile female puberty promoting cues. Our artificial genital touch experiment showed a major effect of genital touch on female sexual maturation. The effects of artificial genital touch on uterine weight (Fig 5I) were even larger than the male effects seen in some of our control experiments (Fig 6K). Thus, our data point to genital touch as a dominant cue for inducing puberty and question the idea that a sensory synergism is required [13]. Our data show that an intact female genital cortex promotes vaginal opening. Thus, the data suggest that genital cortex might be a tactile gateway through which sexual touch promotes female puberty. This question requires further investigation, however, as we did not observe an effect of genital cortex blockade on uterine weight, as described previously in rats [33]. The receptive field properties of genital cortex appear to be tuned to sexual tactile contacts associated with mating [7] and, hence, seem well-suited to relay signals arising from sexual touch. The ensemble of our systemic female hormone application effects on genital cortex growth and our puberty advancing effects established by genital touch leads to speculation about a possible bidirectional connection between genital cortex and the medial preoptic nucleus of the hypothalamus. This region contains gonadotropin releasing hormone (GnRH) neurons whose activity produces high frequency of GnRH release which, in turn, causes gametogenesis and an increase in gonadal steroid hormone secretion [35].

## Materials and methods

### Ethics statement

All experimental procedures were performed according to German guidelines on animal welfare under the supervision of local ethics committees (animal permit numbers: G0193/14 and G0244/16).

### Animals

All experiments were conducted on Wistar rats purchased from Janvier Labs. All animals were kept on a 12 hour to 12 hour, normal light/dark cycle with lights off at 10:00 pm. Rats had ad libitum access to food and water.

### Ovariectomies

Ovariectomies were performed in prepubescent (P21) and adult Wistar rats (P42). For the surgery, animals were anesthetized by injection of an initial dose of 100 mg/kg ketamine and 7.5 mg/kg xylazine. Respiration, blink, and pinch reflex were observed throughout the surgery and, if needed, animals were injected with an extra shot (25%) of ketamine/xylazine mixture (Sigma-Aldrich, St. Louis, MO) or a 25% dose of ketamine (Sigma-Aldrich) alone. Monitoring of temperature was done using a rectal probe and could be maintained with a heating pad (Stoelting, Wood Dale, IL) to 34°C–36°C.

To remove ovaries a small incision was made bilaterally on the animal’s bag. The white fat pad, to which the ovaries are attached to, was pulled out through the body wall. Ovaries were grabbed with forceps and removed from the fallopian tube using small scissors. After successful removal, the white fat pad with fallopian tube was put back through the body wall. The incision was sutured using a self-resorbing thread. The wound was disinfected and looked after in the days following surgery.

### Estradiol treatment

Immature Wistar rats (P26) were divided randomly into groups of 3–4 animals. All animals were weighed daily and the vagina opening was assessed by visual inspection and defined as a complete separation of the membranous sheath covering the vaginal orifice [36]. Photos of clitoris and vagina were taken before the first injection and after the experiment. The assessment of vaginal opening was not blind but the different stages were very clear to detect.

Animals in the testing group were injected subcutaneously every morning with 0.05 μg 17β-estradiol (Sigma-Aldrich), dissolved in 0.4 ml of sesame oil (Sigma-Aldrich), per 100 g body weight. Rats in the control group received the same amount of sesame oil. On day 5 of the treatment, animals were deeply anaesthetized, perfused, and their brains processed, as described below. In addition, uteri of every animal in experiment was taken out while perfusion and weighed.

### Experiments on sexual touch during development

Wistar rats aged P21 were divided into 3 groups. Each animal of group 1 was housed for 9 days together with a sexually experienced adult female rat (approximately P60). Every rat of group 2 was put for 9 days together with a sexually experienced adult male rat (approximately P60). Group 3 rats were also housed together with a sexually experienced adult male rat (approximately P60), but the cages were separated by a wire-mesh. Whereas the animals of group 1 and 2 were able to interact fully with their housing partner, the rats in group 3 could not touch the male they were housed together with. However, they were able to smell, see, and hear the male through the wire-mesh.

Vaginal opening was documented as described above, and pictures of clitoris and vagina were taken before and after the experiment. After 9 days, at the age of P30, animals of all groups were anaesthetized, perfused, and their brains histologically processed as described below. Uteri of all animals were taken out while perfusion and weighed.

### Artificial genital touch

Prepubescent female Wistar rats (P23) were divided into 2 groups. Control animals were placed 3 times for 10 minutes in a small U-shaped arena over 7 days without further treatment and were then returned to their home cage. Animals in the artificial genital touch group were placed 3 times for 10 minutes in a small arena over 7 days, and their clitoris and vulva were contacted with a lubricated brush by a human (female) experimenter (similar to Parada, 2010, [21]). After 7 days (at the age of P30), animals of all groups were anaesthetized, perfused, and their brains histologically processed, as described below. Uteri of all animals were taken out while perfusing and weighed.

### Experiments on blockade of S1 with TTX/Elvax

Animals (P21) were divided into 2 groups. The control group received a chronic Ringer-impregnated Elvax sheet above genital cortex and the rats in the testing group were implanted with a TTX-impregnated Elvax sheet above genital cortex.

Elvax implants were prepared as described before [29, 37, 38]. Briefly Elvax 40p (ethylene-vinyl acetate copolymer; DuPont, Wilmington, DE) was washed for one week in 95% of Ethanol and afterwards dissolved in methylene chloride to obtain a 10% solution. TTX (Abcam, Cambridge, UK) or Ringer (control) was then added in order to achieve a final concentration of 2%. The TTX or Ringer containing Elvax mixture was poured into a glass mold and quickly frozen for 1 hour at −80°C. After 30 minutes, the blocks were removed from the mold and put for another 3 days into the −80°C freezer. On day 3, the Elvax TTX or control blocks were transferred to the −20°C and stored for another 3–4 days. Subsequently, the TTX and Ringer impregnated blocks were cut on a vibratome (Leica, Wetzlar, Germany) into 150 μm sheets, which were cut into 2 mm squares using a scalpel. Before surgical implantation all Elvax sheets were washed for at least 8 hours in distilled water.

For surgical implantation P21 Wistar rats were anesthetized by injection of an initial dose of 100 mg/kg ketamine and 7.5 mg/kg xylazine. Respiration, blink, and pinch reflex were observed throughout the surgery and, if needed, animals were injected with an extra shot (25%) of ketamine/xylazine mixture or a 25% dose of ketamine alone. Monitoring of temperature was done using a rectal probe and could be maintained with a heating pad to 34°C–36°C. Lidocaine was locally injected in the scalp, which was then removed. A craniotomy was performed above genital cortex, and the dura was removed using a bend syringe. The TTX or Ringer impregnated sheets were placed on the brain surface and covered with silicone (Kwik-Cast; World Precision Instruments, Sarasota, FL). The exposed skull was finally covered with dental cement. Animals were allowed to recover from surgery for 2 days. Photos of clitoris and vagina were taken before the behavioral experiment. At the age of P23, implanted animals were put together with a sexually experienced male. After 7 days (at the age of P30), TTX and control animals were anaesthetized and their vaginal opening was documented as described above. Along with the perfusion, the uteri of the animals were cut out and weighed. Brains were histologically processed as described below.

### Histology

At the end of the above-described experiments, animals were anaesthetized using a 20% urethane solution and perfused with phosphate buffer followed by a 2% paraformaldehyde solution (PFA). Brains were removed, hemispheres were separated, and cortices were flattened between 2 glass slides separated by clay spacers [39]. Glass slides were weighed down with small ceramic weights for approximately 3 hours. Afterwards, flattened cortices were stored overnight in 2% PFA and 80 μm sections were cut on a Vibratome (Leica). Sections were stained for cytochrome-oxidase activity using the protocol of Divac et al. [40]. After the staining procedure, sections were mounted on gelatin coated glass slides with Mowiol mounting medium. Subsequently, pictures were taken on an Olympus BX51 microscope and layer 4 areas of S1 were drawn by using a Neurolucida software. S1 maps were reconstructed through serial sections. To exclude experimenter biases, cortical maps were drawn and analyzed by an experimenter blind to the conditions (e.g., estradiol versus sesame oil control group; TTX versus Ringer-treated group).

For the alternating staining procedure with cytochrome c and VGluT2, immunohistochemical labeling was performed using standard procedures. Briefly, brain sections, which should be labeled for VGluT2, were preincubated for an hour at room temperature in a blocking solution (0.1 M PBS, 2% bovine serum albumin, and 0.5% Triton X-100). Afterwards, primary antibodies were diluted in a solution containing 0.5% Triton X-100 and 1% bovine serum albumin. The primary antibody against VGluT2 was incubated with the free-floating sections for at least 24 hours under mild shaking at 4°C. Incubations with the primary antibody was followed by detection with a secondary antibody coupled to the fluorophore Alexa 488. The secondary antibody was diluted (1:500) in 0.5% Triton X-100 and the reaction was allowed to proceed for 2 hours in the dark at room temperature. After the staining procedure, sections were mounted on gelatin coated glass slides with Mowiol mounting medium.

### Quantification of somatosensory areas and sizes

The area of various somatosensory regions was measured by outlining the anatomical region of interest and calculating its area using Neurolucida area calculating tool. The area of the following cortical representations was measured: hindpaw, forepaw, trunk, interlimb cortex, and clitoris. The fraction of genital cortex of the whole S1 area was calculated by dividing the clitoris area by the value of the S1 area. The same was done for the fraction of genital cortex of the interlimb cortex. All statistic tests were conducted in Matlab.

### Scoring of vaginal opening

In order to quantify the state of vaginal opening after the above described experiments, we assigned different states with vaginal opening scores. A score of 0 points to a closed vagina, whereas a score of 0.5 describes a vagina, which was about to open. Finally, a score of 1 represents an open vagina.

## Supporting information

S1 Fig Micrographs showing the pubertal expansion of genital cortex, but not its maintenance in adults requires sex hormones. Figure S1 related to Fig 1: Micrographs showing the pubertal expansion of genital cortex, but not its maintenance in adults requires sex hormones. **A**, Upper panel: Tangential section through S1 of a P25 old animal stained for cytochrome oxidase activity. Lower panel: The corresponding map drawn from the section shown above, genital cortex shown in black.**B**, Same as A, but the section and map stems from an animal aged P42. The genital cortex is larger than in the P25 animal. **C**, Same as A for an animal aged P42, which was ovariectomized before puberty at P20. Note that the size of the genital cortex is comparable to the genital cortex of the youngfemale shown in A. **D**, Same as A for an animal aged P60, which was ovariectomized after puberty at P42. The clitoris representation is greater than in the young ovariectomized animal (C) but comparable in size to the P42 aged animal (B).
(TIF)

S2 Fig Comparison of normalization of genital cortex to primary somatosensory cortex (S1) and posteromedial barrel subfield (PMBSF). Figure S2 related to Fig 1: Comparison of normalization of genital cortex to primary somatosensory cortex (S1) and posteromedial barrel subfield (PMBSF).**A**, Fraction of genital cortex of the entire S1 in hemispheres of P25, P42 females and females which were ovariectomized at either P20 or P42. **B**, Same as A but fraction of genital cortex of the PMBSF is shown. Note that the same effects are seen as in A, namely a substantial growth of the genital cortex between P25 and P42 animals. Female rats ovariectomized during prepuberty had smaller genital cortices than animals ovariectomized after puberty. Graphically distribution looks similar in A and B and no big difference in the variability can be make out. See also S1 Table. **C**, Absolute area of PMBSF in hemispheres of P25, P42, in prepuberty (P20) ovariectomized and postpuberty (P42) ovariectomized female rats. See also S1 Data.
(TIF)

S3 Fig Micrographs showing systemic estradiol application drives genital cortex growth and advances puberty. Figure S3 related to Fig 2: Micrographs showing systemic estradiol application drives genital cortex growth and advances puberty.**A**, Upper panel: Tangential section through S1 of a hemisphere obtained from an animal which received subcutaneous sesame oil injections over 5 days. The section was stained for cytochrome c and shows the reconstructed clitoris area best. Lower panel: Corresponding reconstructed map from the section plotted above. **B**, Same as A, but the section and drawn map are obtained from an animal which received daily estradiol injections. Note that the clitoris area is greater compared to the example section and map shown in A.
(TIF)

S4 Fig Genital cortex growth is due to the invasion of dysgranular territories by putative genital thalamic afferents. Figure S4 related to Fig 3: Genital cortex growth is due to the invasion of dysgranular territories by putative genital thalamic afferents.**A**, Upper, tangential section through S1 of an animal aged P14. The section was stained for cytochrome oxidase activity. Lower, map of the somatosensory areas drawn on the section shown above. **B**, Upper, adjacent tangential section through S1 obtained from the same hemisphere, which is shown in A. The section was stained with antibodies (green fluorescence) against VGluT2 (vesicular glutamate transporter 2), which is expressed in thalamocortical afferents. Lower, map of the somatosensory areas drawn on the section shown above. Note that the reconstructed areas correspond in size when comparing A and B.
(TIF)

S5 Fig Genital cortex size and uterine weight. Figure S5 related to Figs 5 & 6: Genital cortex size and uterine weight.**A**, Genital cortex size is plotted against uterine weight for all experiments. No tight relationship can be observed. Note that a large uterine weight (≥ 0.15 g) is rarely associated with a small (≤ 1%) relative genital cortex size.**B**, Same as A, but for specific experimental conditions See also S1 Data.
(TIF)

S1 Table Table S1 related to Fig 1: Variation coefficient. (TIF)

S1 Data All supporting data. Individual data for Figs 1, 2, 3, 4, 5 and 6 and S2 and S5 Figs.
(XLSX)

## Abbreviations

GnRH: gonadotropin releasing hormone
P: postnatal day
PMBSF: posteromedial barrel subfield
S1: somatosensory cortex
TTX: tetrodotoxin
VGluT2: vesicular glutamate transporter 2
