# Supplementary figures and images for: Development of rat female genital cortex and control of female puberty by sexual touch
Source: PLoS Biol. 2017 Sep 21;15(9):e2001283. doi: 10.1371/journal.pbio.2001283 (PMC5608169; doi:10.1371/journal.pbio.2001283)

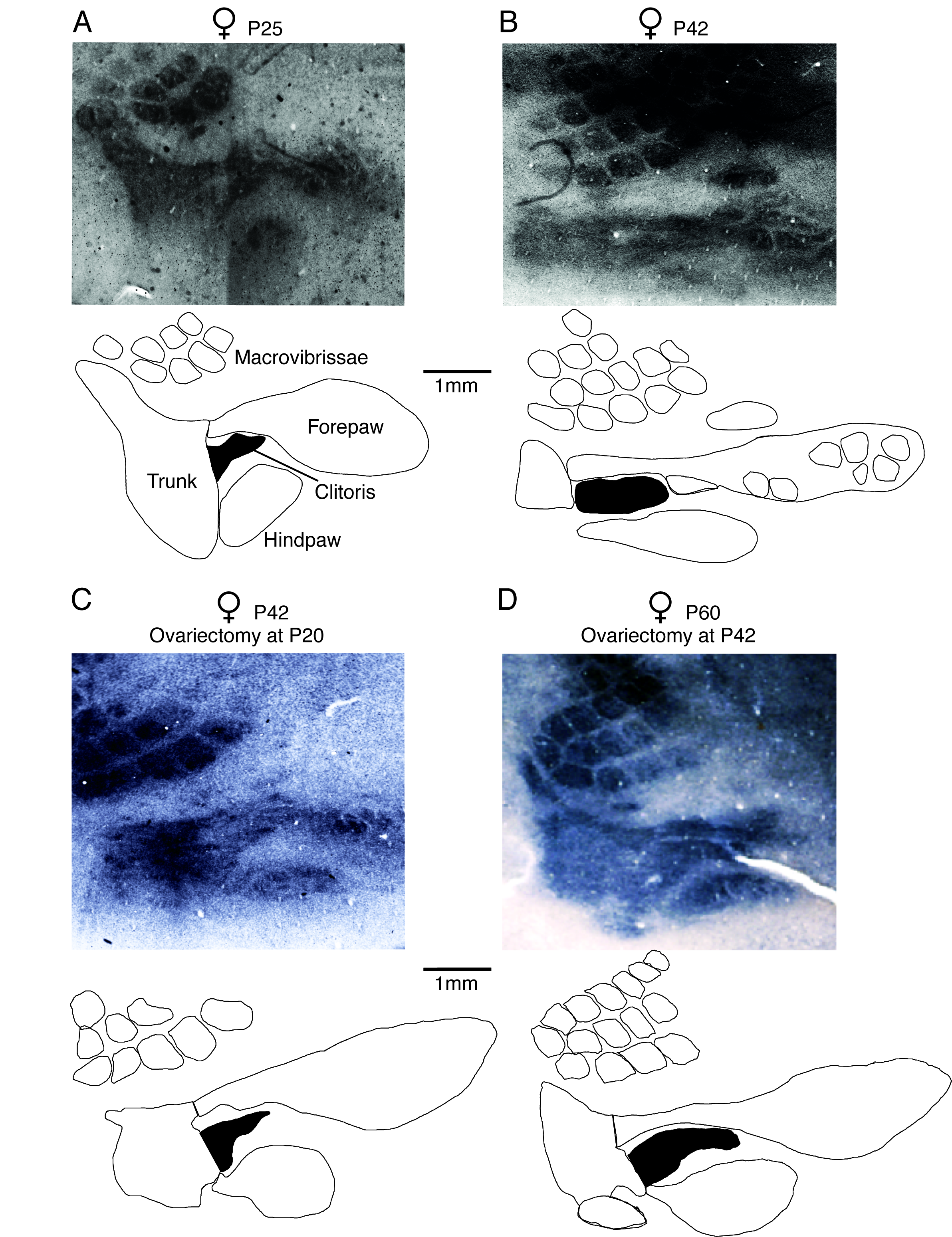

Supplement: S1 Fig — Figure S1 related to Fig 1: Micrographs showing the pubertal expansion of genital cortex, but not its maintenance in adults requires sex hormones. A, Upper panel: Tangential section through S1 of a P25 old animal stained for cytochrome oxidase activity. Lower panel: The corresponding map drawn from the section shown above, genital cortex shown in black.B, Same as A, but the section and map stems from an animal aged P42. The genital cortex is larger than in the P25 animal. C, Same as A for an animal aged P42, which was ovariectomized before puberty at P20. Note that the size of the genital cortex is comparable to the genital cortex of the youngfemale shown in A. D, Same as A for an animal aged P60, which was ovariectomized after puberty at P42. The clitoris representation is greater than in the young ovariectomized animal (C) but comparable in size to the P42 aged animal (B). (TIF) [file pbio.2001283.s001.tif]

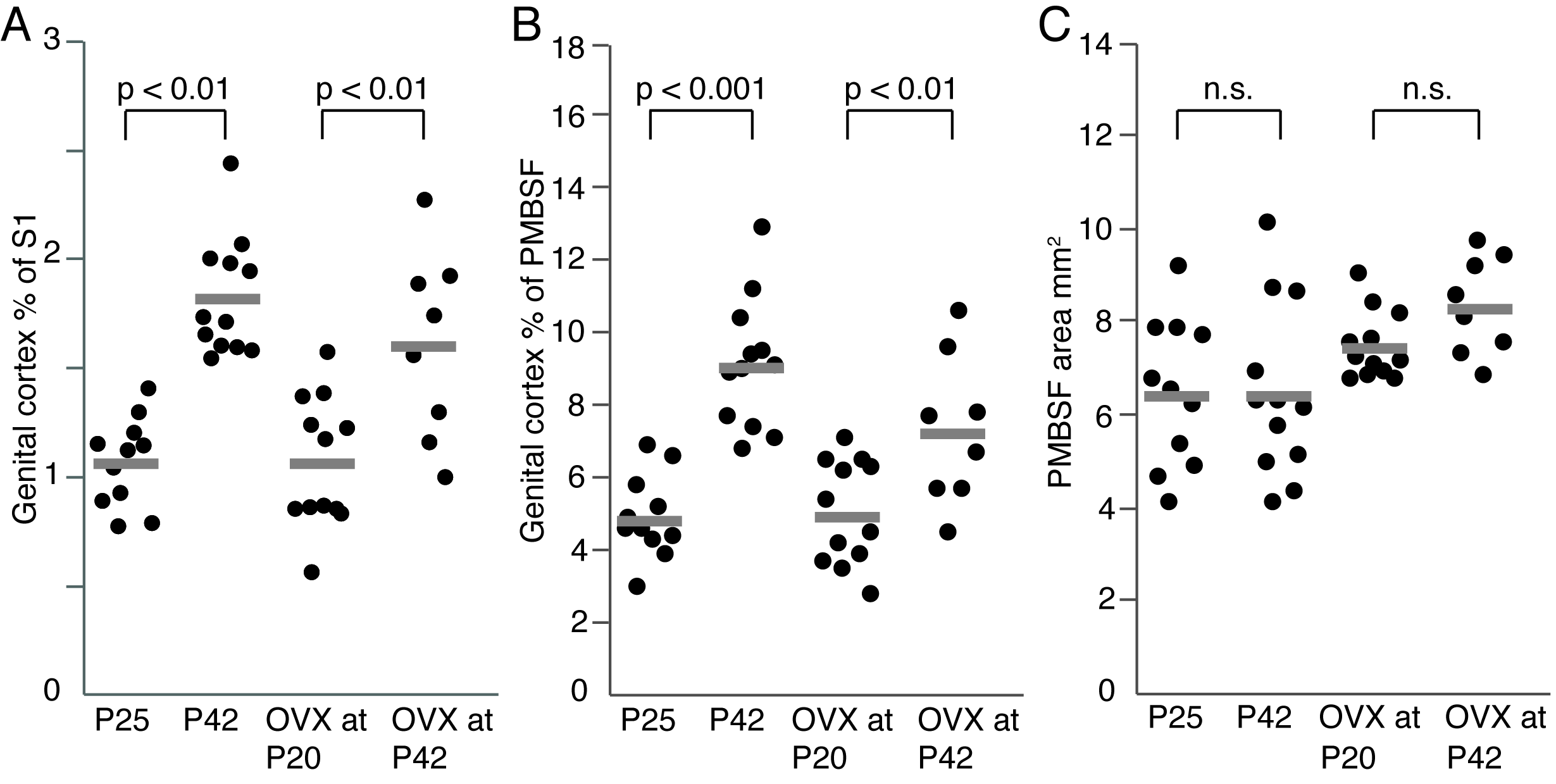

Supplement: S2 Fig — Figure S2 related to Fig 1: Comparison of normalization of genital cortex to primary somatosensory cortex (S1) and posteromedial barrel subfield (PMBSF).A, Fraction of genital cortex of the entire S1 in hemispheres of P25, P42 females and females which were ovariectomized at either P20 or P42. B, Same as A but fraction of genital cortex of the PMBSF is shown. Note that the same effects are seen as in A, namely a substantial growth of the genital cortex between P25 and P42 animals. Female rats ovariectomized during prepuberty had smaller genital cortices than animals ovariectomized after puberty. Graphically distribution looks similar in A and B and no big difference in the variability can be make out. See also S1 Table. C, Absolute area of PMBSF in hemispheres of P25, P42, in prepuberty (P20) ovariectomized and postpuberty (P42) ovariectomized female rats. See also S1 Data. (TIF) [file pbio.2001283.s002.tif]

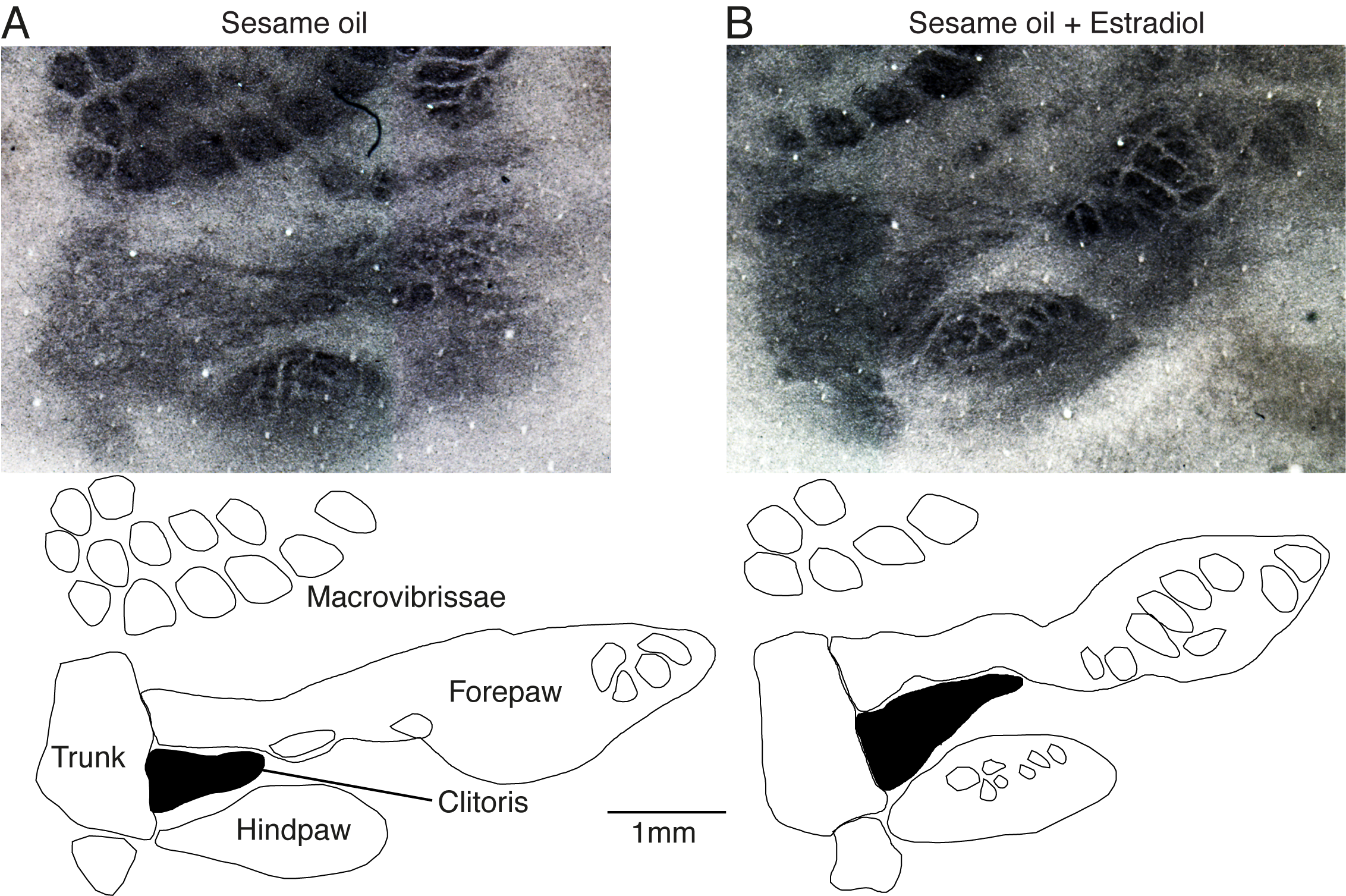

Supplement: S3 Fig — Figure S3 related to Fig 2: Micrographs showing systemic estradiol application drives genital cortex growth and advances puberty.A, Upper panel: Tangential section through S1 of a hemisphere obtained from an animal which received subcutaneous sesame oil injections over 5 days. The section was stained for cytochrome c and shows the reconstructed clitoris area best. Lower panel: Corresponding reconstructed map from the section plotted above. B, Same as A, but the section and drawn map are obtained from an animal which received daily estradiol injections. Note that the clitoris area is greater compared to the example section and map shown in A. (TIF) [file pbio.2001283.s003.tif]

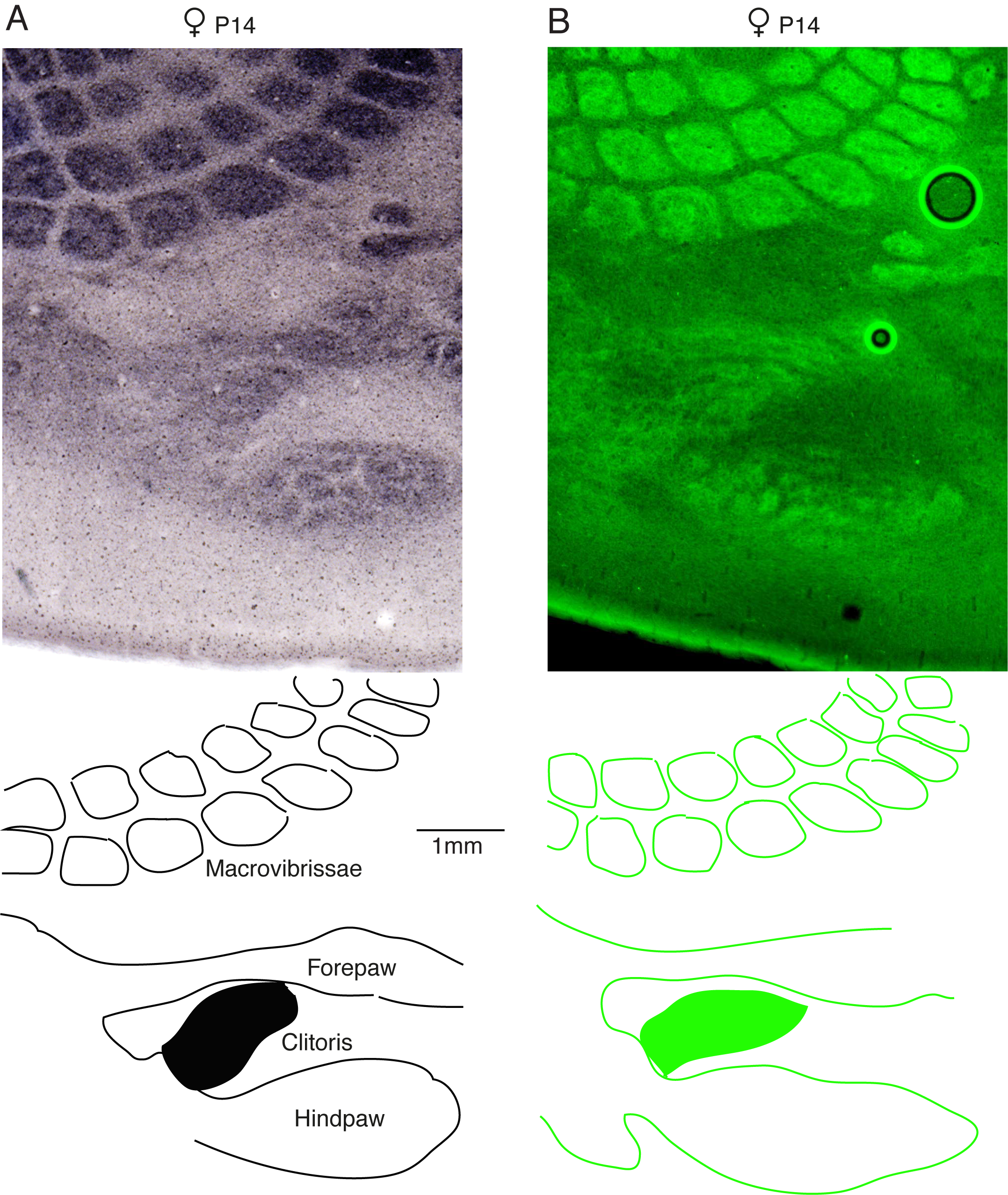

Supplement: S4 Fig — Figure S4 related to Fig 3: Genital cortex growth is due to the invasion of dysgranular territories by putative genital thalamic afferents.A, Upper, tangential section through S1 of an animal aged P14. The section was stained for cytochrome oxidase activity. Lower, map of the somatosensory areas drawn on the section shown above. B, Upper, adjacent tangential section through S1 obtained from the same hemisphere, which is shown in A. The section was stained with antibodies (green fluorescence) against VGluT2 (vesicular glutamate transporter 2), which is expressed in thalamocortical afferents. Lower, map of the somatosensory areas drawn on the section shown above. Note that the reconstructed areas correspond in size when comparing A and B. (TIF) [file pbio.2001283.s004.tif]

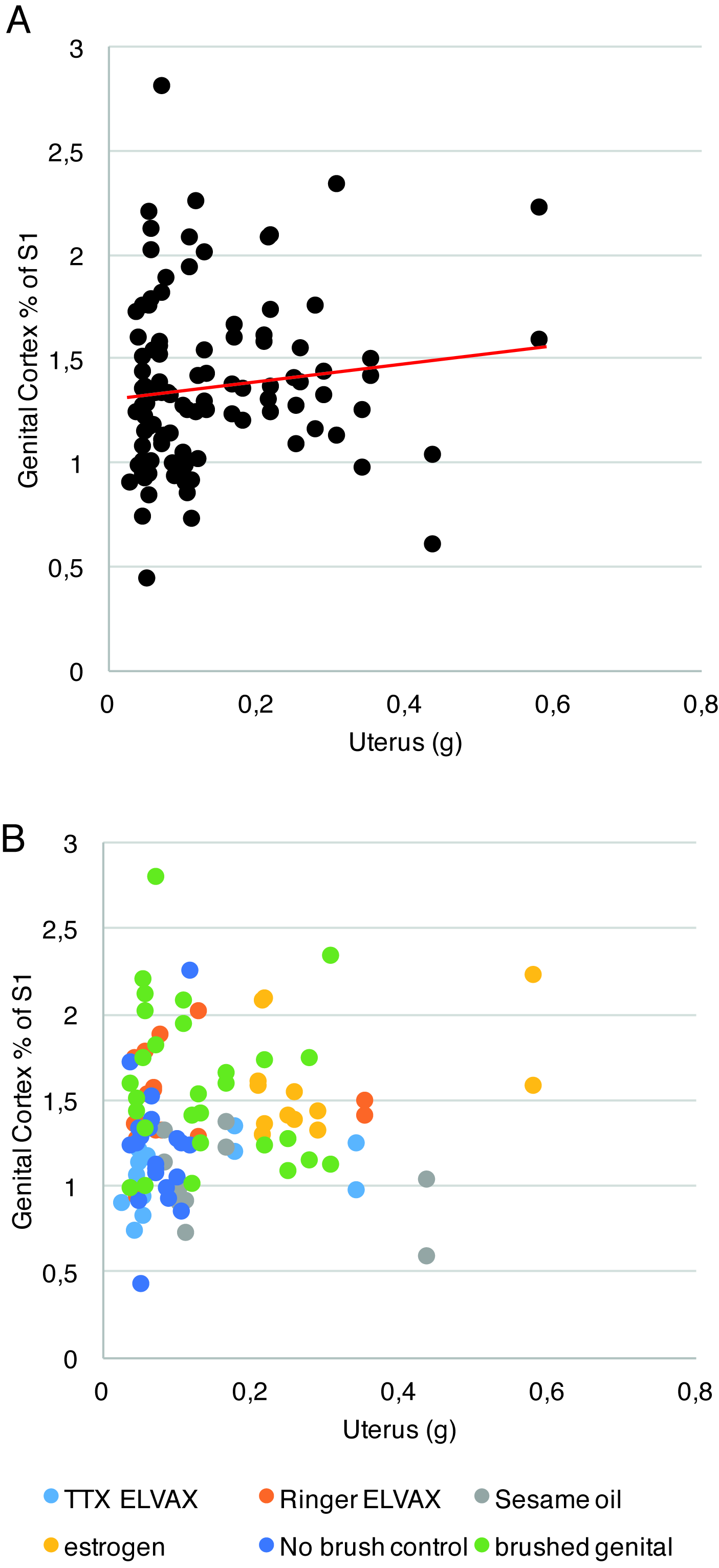

Supplement: S5 Fig — Figure S5 related to Figs 5 & 6: Genital cortex size and uterine weight.A, Genital cortex size is plotted against uterine weight for all experiments. No tight relationship can be observed. Note that a large uterine weight (≥ 0.15 g) is rarely associated with a small (≤ 1%) relative genital cortex size.B, Same as A, but for specific experimental conditions See also S1 Data. (TIF) [file pbio.2001283.s005.tif]

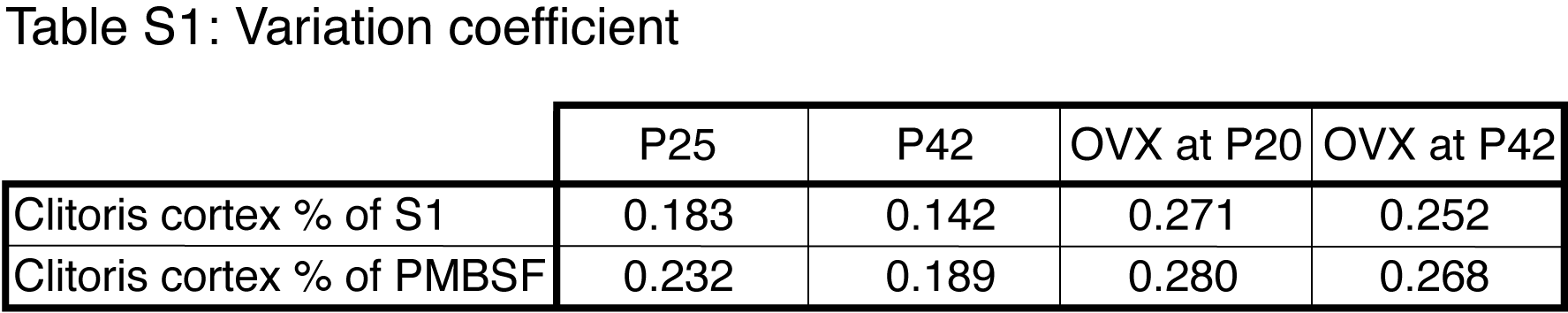

Supplement: S1 Table — (TIF) [file pbio.2001283.s006.tif]
